# Rapid Primary Care Follow-up from the ED to Reduce Avoidable Hospital Admissions

**DOI:** 10.5811/westjem.2017.5.33593

**Published:** 2017-07-14

**Authors:** Amanda S. Carmel, Peter Steel, Robert Tanouye, Aleksey Novikov, Sunday Clark, Sanjai Sinha, Judy Tung

**Affiliations:** * Weill Cornell Medical College, Department of Medicine, New York, New York; † Weill Cornell Medical College, Department of Emergency Medicine, New York, New York

## Abstract

**Introduction:**

Hospital admissions from the emergency department (ED) now account for approximately 50% of all admissions. Some patients admitted from the ED may not require inpatient care if outpatient care could be optimized. However, access to primary care especially immediately after ED discharge is challenging. Studies have not addressed the extent to which hospital admissions from the ED may be averted with access to rapid (next business day) primary care follow-up. We evaluated the impact of an ED-to-rapid-primary-care protocol on avoidance of hospitalizations in a large, urban medical center.

**Methods:**

We conducted a retrospective review of patients referred from the ED to primary care (Weill Cornell Internal Medicine Associates – WCIMA) through a rapid-access-to-primary-care program developed at New York-Presbyterian / Weill Cornell Medical Center. Referrals were classified as either an avoided admission or not, and classifications were performed by both emergency physician (EP) and internal medicine physician reviewers. We also collected outcome data on rapid visit completion, ED revisits, hospitalizations and primary care engagement.

**Results:**

EPs classified 26 (16%) of referrals for rapid primary care follow-up as avoided admissions. Of the 162 patients referred for rapid follow-up, 118 (73%) arrived for their rapid appointment. There were no differences in rates of ED revisits or subsequent hospitalizations between those who attended the rapid follow-up and those who did not attend. Patients who attended the rapid appointment were significantly more likely to attend at least one subsequent appointment at WCIMA during the six months after the index ED visit [N=55 (47%) vs. N=8 (18%), P=0.001].

**Conclusion:**

A rapid-ED-to-primary-care-access program may allow EPs to avoid admitting patients to the hospital without risking ED revisits or subsequent hospitalizations. This protocol has the potential to save costs over time. A program such as this can also provide a safe and reliable ED discharge option that is also an effective mechanism for engaging patients in primary care.

## INTRODUCTION

Hospital admissions from the emergency department (ED) now account for approximately 50% of all admissions.^1^ At the same time the Centers for Medicare & Medicaid Services restructured federal policies in 2013, specifically the Two-Midnight Rule and the revised 30-day readmission penalties, to encourage hospital systems to reduce short-stay admissions.^2^ The majority of ED visits leading to hospital admissions are classified as intermediate severity.^3^ Intermediate severity cases include exacerbations of chronic diseases (e.g. congestive heart failure) and acute presentations of complex medical illnesses (e.g. hyperglycemia in diabetics).^3^ Some ED admissions may not benefit from inpatient care if outpatient care can be optimized. Further, discontinuity between ED care and outpatient care can lead to over-testing and conflicting care plans, as well as less-effective preventive care and chronic disease management.^4^–^6^ Thus, ensuring rapid access to outpatient care presents a potentially high-yield intervention with the goal of reducing admissions, repeated ED visits and improving chronic disease management.

The decision to admit a patient from the ED is complex. The disposition of ED patients takes into account not only the clinical scenario but also the presence or absence of immediate and reliable outpatient follow-up. Evidence suggests that access to primary care after ED discharge is critical and challenging.^2^–^5^,^8^–^11^ Further, patients’ perceptions of their inability to access timely follow-up is a primary motivator for return ED visits and readmissions.^12^–^15^ This may be of particular significance in the publicly insured; as many as two thirds of Medicaid patients with urgent conditions may be unable to procure timely appointments after an ED visit.^16^

Research on successful programs to facilitate care transitions between the ED and primary care is limited.^17^ Case management and patient navigation are the only interventions found to consistently reduce ED visits and increase primary care follow-up.^9^,^18^–^20^ However, to our knowledge there are no studies that directly evaluate the extent to which hospital admissions from the ED may be averted with access to rapid (next business day) primary care follow-up. In this study we evaluate the impact of an ED-to-rapid-primary-care follow-up protocol on avoidance of hospitalizations in a large, urban medical center. As secondary objectives we also determined the rates of rapid follow-up appointment completion, ED revisits, hospitalizations, and subsequent primary care engagement among patients referred through this protocol.

## METHODS

In May 2014, an ED-to-rapid-primary-care protocol was designed and initiated through the collaboration of the New York-Presbyterian Hospital (NYP), Weill Cornell Emergency Department and Weill Cornell Internal Medicine Associates (WCIMA), a large academic faculty and resident practice at the NYP / Weill Cornell campus. This protocol uses a “transitions team” (a registrar and a nurse care manager at WCIMA), as well as a secure Intranet-based electronic and telephone scheduling system to facilitate the rapid scheduling of ED patients with a primary care provider at WCIMA. The system was created for emergency physicians (EP) to use for patients who could avoid an admission to the hospital if given rapid primary care follow-up (within 24 hours on weekdays or a Monday appointment for those seen over the weekend). Since the beginning of this program, each patient referred through this process has been tracked for quality and safety purposes via a secure registry kept by the WCIMA transitions team. Although the protocol was created primarily to serve those patients for whom an admission could be averted, our registry demonstrated a diverse set of reasons for the ED referrals. For this reason, our chart reviewers were asked to isolate referrals that potentially represented an avoided admission.

Population Health Research Capsule

What do we already know about this issue?

Access to primary care after emergency department (ED) discharge is critical and challenging. Research on programs to facilitate care transitions between the ED and primary care is limited.

What was the research question?

To what extent can hospital admissions from the ED be averted with access to rapid (next business day) primary care follow-up?

What was the major finding of the study?

Rapid access to primary care is a safe alternative to low-acuity admissions and engages patients into primary care.

How does this improve population health?

Efforts to reduce low-acuity hospitalizations can include, for select patients, rapid access to primary care. Rapid access may also offer an opportunity to engage such patients in primary care.

We conducted a retrospective cohort study of all patients referred for rapid follow-up through this protocol from May 2014 to May 2015 – the first year of the program. Data collected from the electronic medical record (EMR) review included demographic information, primary ED discharge diagnosis, ED visit level of service, rapid-primary-care appointment completion, outpatient visit level of service, 72-hour, 30-day, and six-month ED revisits, 30-day and six-month hospitalization, and mortality. Engagement in primary and/or specialty care was also assessed and was defined as completing at least one additional appointment in the six months following the rapid primary care appointment. The protocol was approved by the Weill Cornell Medical College Institutional Review Board.

We were interested in identifying and studying those patients for whom this protocol could most benefit (through the avoidance of a hospitalization) or potentially harm (through an ED discharge without completing a rapid appointment). To identify the first subset, physician reviewers were asked to review the patient charts and assess whether the referral represented an “avoided admission” by answering the following hypothetical question:

Without the option to refer this patient for rapid follow-up to WCIMA:

1. I definitely would have admitted this patient
2. I might have admitted this patient
3. I probably would not have admitted this patient
4. I definitely would not have admitted this patient.

Because internal medicine (IM) physicians and EPs may have disparate practices or thresholds for admitting patients, we had abstractors from both disciplines review the EMRs of each subject. This was done to enhance generalizability for institutions that have admitting internists instead of admitting EPs.

There were three IM physician reviewers. To establish agreement, the IM reviewers analyzed the same charts until at least 90% agreement was reached. Scores 1 and 2 were considered “would have admitted,” and 3 and 4 were considered “would not have admitted.” Once at least 90% agreement was reached, the remaining charts were divided evenly among the IM reviewers. Two EM reviewers used the same approach for reviewing charts. Both an IM and an emergency physician reviewed all charts. In recognition of potential hindsight bias, reviewers were instructed to only review EMR notes and data from the day of the ED visit or prior to the visit. Encounters or data that occurred after the ED discharge were not considered. The reviewers were blinded to the assessments of their colleagues.

We analyzed the characteristics and outcomes of patients in two ways. The first analysis included patients who were considered by the EM reviewers to represent an avoided admission. Although we compared categorizations between the IM and EM reviewers, we used the EM determination because in our institution, decisions to admit are made by EPs. Our second analysis compared characteristics and outcomes of patients who did and did not attend their rapid appointment.

Finally, we conducted a subgroup analysis among patients for whom this protocol could potentially have harmed – those patients discharged from the ED and who did not arrive for their rapid follow-up appointment. We described their characteristics and outcomes and performed a more in-depth, qualitative chart review of these patients.

### Statistical Analysis

We performed all analyses using Stata 14.0 (Stata Corp., College Station, TX). Data are presented as proportions, means with standard deviations (SDs), and medians with interquartile ranges (IQRs). Analyses were done using chi-square, Fisher’s exact test, Student’s t-test, and Kruskal-Wallis test, as appropriate. All *P* values are two-tailed, with *P*<0.05 considered statistically significant.

## RESULTS

We reviewed the charts of 162 subjects referred for rapid follow-up at WCIMA from the ED between May 22, 2014, and May 27, 2015. The subjects had a median age of 49 (IQR 33 – 63) years and 59% were female; 45% had commercial insurance, 14% were insured with Medicare, 32% with Medicaid, and 9% had no insurance. Most of the subjects were new to WCIMA, 114 (70%), and among these 20% had an outside PCP, 45% did not have an outside PCP, and 35% had no documentation about an outside PCP.

Nearly three-quarters of subjects had an ED level of service of 4 or 5. The top three categories for reasons for a referral for rapid WCIMA follow-up were gastroenterology, such as follow-up of abdominal pain (N=26, 16%), need to establish primary care (N=26, 16%), and cardiology, such as hypertension follow-up (N=23, 14%).

When the 4-point avoidability scale was collapsed into binary categories (referral represented an avoided admission or referral did not represent an avoided admission), agreement between the two physician groups was high at 75.93% (P<0.001). Isolating the referrals that were considered avoided admissions, EPs classified 26 (16%) of referrals compared with IM physicians who classified 43 (27%) as avoided admissions.

Of the 162 patients referred for rapid follow-up, 118 (73%) arrived for their rapid appointment, 31 (19%) did not arrive for their appointment, 9 (6%) cancelled, 2 (1%) declined, and 2 (1%) were unable to be contacted to make the appointment.

Characteristics of patients classified by EPs as having an avoided admission compared to those not considered to be among the avoided admissions are shown in Table 1. Patients with avoided admissions were older than those without avoided admissions. This group was also more likely to have a higher ED level of service. There were no statistically significant differences between the avoided admission and not-avoided admissions groups with respect to arrival to the rapid-primary-care appointment. Subsequent ED visits (at 72 hours, 30 days, and six months) and hospitalizations (at 30 days and six months) were similar between the groups. Primary care engagement following the index ED visit and referral for rapid primary care follow-up was also similar.

Characteristics of patients who attended their rapid primary care follow-up appointment compared to those who did not are shown in Table 2. Patients who attended their appointments and those who did not were similar with respect to most demographic characteristics. Those who attended were more likely to not have an outside primary care physician (PCP). Classification as an avoided admission, subsequent ED visits (at 72 hours, 30 days, and six months), and subsequent hospitalizations (at 30 days and six months) were similar between the groups. Primary care engagement differed significantly between the two groups with those attending their rapid follow-up appointment more likely to engage with primary care in the six months following the index ED visit.

None of the subjects who arrived for the rapid follow-up appointment were sent back to the ED from that appointment. Based on our chart review, there were no deaths among the entire patient cohort within the six months following the index ED discharge.

Patients who were new to WCIMA were less likely to be engaged in primary care during the six months after the index ED visit at WCIMA (31% vs. 58%; P=0.001). Patients without an outside PCP were more likely than those with an outside PCP, or those for whom it was not known whether they had an outside PCP, to engage in any primary care during the six months after the index ED visit (43% vs. 30% vs. 20%; P=0.06).

We considered nine (6%) patients to be at highest risk for adverse outcomes because they were considered an avoided admission and did not attend their rapid follow-up appointment (Figure). Most (78%) did complete an outpatient follow-up visit despite missing their rapid appointment; these visits occurred on average within 1–2 weeks of the ED index visit. None of the eight patients returned to our ED or had a subsequent admission to our hospital.

## DISCUSSION

This retrospective review of the first year of an ED-to-rapid-primary-care follow-up protocol offers a number of points for discussion related to quality, safety, and engagement of ED patients discharged to primary care. In this study we aimed to evaluate the extent to which the option to refer a patient for rapid follow-up would impact the EP’s decision to admit a patient. While many previous studies have reported on defining “preventable” admissions and re-admissions, to our knowledge there is not a standard definition of admission avoidability.^9^ To assess the extent to which the referrals represented an avoided admission, we developed an avoidability rating scale to isolate patients referred for this reason from the ED. Based on this avoidability assessment score and as determined by the EP reviewers, 16% of all subjects referred represented an avoided admission. While the overall number of patients in the first year of our program was small, over time this could represent a substantial cost savings. Furthermore, safely avoiding a hospitalization removes a significant burden on both patients and the hospital system.

An additional aim of our program was to provide the option for reliable and accessible rapid primary care follow-up for patients being discharged from ED. The majority of all subjects in the entire cohort as well as in the avoided admission group arrived for their rapid follow-up appointment. This rate is higher than the average appointment completion rate at our clinic and in other reports on completion of rapid follow-up after ED discharge.^5^ We postulate this may reflect the timing of the appointment offering as well as communication from the ED providers around the importance of the follow-up. Thus, in most cases the opportunity to refer a patient for rapid primary care follow-up at WCIMA represented a safe and reliable discharge plan. None of the subjects were sent back to the ED from the rapid follow-up appointment, which suggests that at the time of follow-up there was no indication for emergency or inpatient care.

However, nine subjects in the avoided admissions group did not arrive for their rapid follow-up appointment. This group could be considered at the highest risk for an unsafe outcome since they likely would have been admitted without the existence of the rapid follow-up appointment. Fortunately, seven out of the nine subjects (78%) did complete outpatient follow-up with either primary or specialty care within 1–2 weeks of the ED discharge, suggesting that outpatient referral was successful and that more flexible timing of the rapid appointment should be considered. Two out of the nine were lost to follow-up: a 30-year-old female who was newly diagnosed with diabetes, and a 35-year-old female with congenital heart disease and atypical chest pain. None of the nine subjects returned to our ED or was admitted to our hospital within six months.

Ten percent of subjects in this cohort revisited the ED within 30 days of the index ED visit. This is lower than the national 30-day ED revisit rate of 19.9% reported by the Healthcare Cost and Utilization Project.^10^ The rate in this cohort was comparable to historical ED revisit rates at our own institution (8%). These comparisons further indicate the safety and efficiency of the program.

Anecdotal evidence as well as some observational studies suggest that EPs may be more likely to admit patients to the hospital than their IM colleagues or admit patients who could be safely discharged from the ED.^9^ However, in our retrospective review EPs rated fewer patients as those they would have admitted than the IM physicians.

The clinical reasons for referral for rapid follow-up in this cohort were diverse, which makes it challenging to identify particular diagnoses that might be especially appropriate for this program. However, the need to establish primary care was one of the most common reasons for referral. This reinforces the findings noted below that the ED encounter offers an opportunity to engage patients in primary care.

Receiving regular primary care is associated with a number of health benefits including increased receipt of preventative services and better chronic disease management.^11^–^15^ A large percentage of subjects in our cohort engaged in primary care after the rapid primary care referral. Furthermore, subjects who attended the rapid follow-up appointment were significantly more likely to engage in primary care, suggesting that right after an ED visit may be an optimal time to capture patients into regular primary care.

## LIMITATIONS

There are a number of limitations to this study. Our primary aim was to assess avoidability. Since no standard avoidability criteria exist, we were required to devise our own assessment. Therefore, we cannot ensure the validity or reproducibility of this assessment scale. Further, the retrospective nature of our evaluation cannot completely simulate the patient-care interaction where the actual admission decision was made, so it may be open to biases and is subjective. In addition, while we were able to collect data on follow-up, hospitalizations, and ED revisits at our own institution, as well as mortality data based on our EMR review, we were not able to include data on hospitalizations, ED visits, or outpatient follow-up at other institutions and could not verify mortality data in all cases. The average age of the patients in this sample was 50; thus, our findings may not be applicable to an older population. However, our results suggest that younger patients may be good candidates for a program such as this. Finally, this study was conducted at a single institution, which may limit generalizability. Further, since this is an analysis of the first year of the program only, we had a relatively small number of subjects.

## CONCLUSION/FUTURE DIRECTIONS

Results from this analysis suggest that a protocol to ensure rapid primary care follow-up for ED patients can allow emergency physicians to avoid some patient admissions. Such a program has the potential for cost savings over time given that, in general, outpatient care often represents a cost savings when compared to an inpatient admission. In the future we intend to conduct a cost analysis to compare the inpatient costs saved by an avoided admission with those incurred from outpatient follow-up and from reserving appointment slots for the rapid discharge program. We also hope to conduct a prospective study. Our data suggest that a rapid-ED-to primary-care follow-up program can provide a safe and reliable ED discharge option that is also an effective mechanism for engaging patients in primary care. Such primary care engagement has the potential to lead to further containment in overall healthcare costs, as well as to improved patient care and health outcomes.

## Figures and Tables

**Figure:**
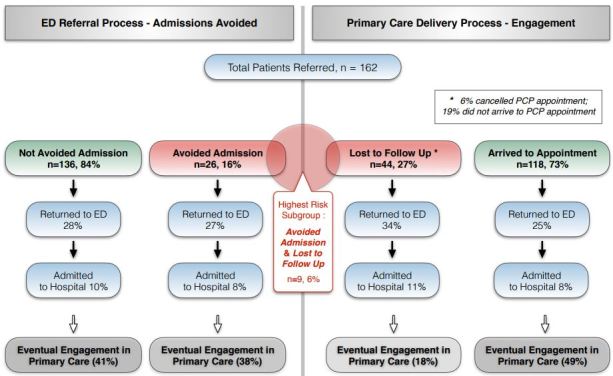
Patients referrals and outcomes. *ED*, emergency department; *PCP*, primary care physician.

**Table 1:** Characteristics of patients with and without avoided admissions due to rapid primary care follow-up, as defined by emergency physicians.

	Overall (n=162)	Admission avoided (n=26)	No avoided admission (n=136)	p value
Demographic characteristics				
Age (years), median (IQR)	50 (33 – 63)	55 (42 – 74)	49 (31 – 60)	0.02
Female	95 (59%)	15 (58%)	80 (59%)	0.92
Insurance				0.20
Commercial	73 (45%)	10 (38%)	63 (46%)	
Medicare	23 (14%)	6 (23%)	17 (13%)	
Medicaid	52 (32%)	6 (23%)	46 (34%)	
None	14 (9%)	4 (15%)	10 (7%)	
ED level of service				0.04
2	4 (2%)	0 (0%)	4 (3%)	
3	41 (25%)	2 (8%)	39 (29%)	
4	94 (58%)	17 (65%)	77 (57%)	
5	23 (14%)	7 (27%)	16 (12%)	
Patient new to Weill Cornell Internal Medicine Associates (WCIMA)	114 (70%)	17 (65%)	97 (71%)	0.54
Patient has outside primary care physician (PCP) (n=114)				0.35
Yes	23 (20%)	5 (29%)	18 (19%)	
No	51 (45%)	5 (29%)	46 (47%)	
Unknown	40 (35%)	7 (41%)	33 (34%)	
Outcomes				
Arrived for rapid follow-up	118 (73%)	17 (65%)	101 (74%)	0.35
Return emergency department (ED) visit within 72 hours index ED visit	7 (4%)	1 (4%)	6 (4%)	1.00
ED visit within 30 days of index ED visit	16 (10%)	2 (8%)	14 (10%)	1.00
ED visit within 6 months of index ED visit	45 (28%)	7 (27%)	38 (28%)	0.92
Hospitalization within 30 days of index ED visit	7 (4%)	1 (4%)	6 (4%)	1.00
Hospitalization within 6 months of index ED visit	15 (9%)	2 (8%)	13 (10%)	1.00
Primary care engagement				
With WCIMA during 6 months after index ED visit	63 (39%)	10 (38%)	53 (39%)	1.00
With WCIMA during 6 months after initial rapid follow-up appointment (n=118)	55 (47%)	7 (41%)	48 (48%)	0.79
With another PCP during 6 months after index ED visit	4 (2%)	0 (0%)	4 (3%)	1.00
With WCIMA or another PCP during 6 months after index ED visit	66 (40%)	10 (38%)	56 (41%)	0.80

**Table 2:** Characteristics of patients according to attending rapid primary care appointment after emergency department discharge.

	Attended rapid appointment (n=118)	Did not attend rapid appointment (n=44)	p value
Demographic characteristics			
Age (years), median (IQR)	50 (37 – 65)	46 (31 – 62)	0.79
Female	73 (62%)	22 (50%)	0.17
Insurance			0.71
Commercial	51 (43%)	22 (50%)	
Medicare	19 (16%)	4 (9%)	
Medicaid	38 (32%)	14 (32%)	
None	10 (8%)	4 (9%)	
ED level of service			0.28
2	2 (2%)	2 (5%)	
3	30 (25%)	11 (25%)	
4	72 (61%)	22 (50%)	
5	14 (12%)	9 (20%)	
Patient new to Weill Cornell Internal Medicine Associates (WCIMA)	84 (71%)	30 (68%)	0.71
Patient has outside primary care physician (PCP) (n=114)			0.03
Yes	17 (20%)	6 (20%)	
No	43 (51%)	8 (27%)	
Unknown	24 (29%)	16 (53%)	
Outcomes			
Emergency physician classification as avoided admission	17 (14%)	9 (20%)	0.35
Return emergency department (ED) visit within 72 hours index ED visit	4 (3%)	3 (7%)	0.39
ED visit within 30 days of index ED visit	10 (8%)	6 (14%)	0.33
ED visit within 6 months of index ED visit	30 (25%)	15 (34%)	0.27
Hospitalization within 30 days of index ED visit	6 (5%)	1 (2%)	0.68
Hospitalization within 6 months of index ED visit	10 (8%)	5 (11%)	0.57
Primary care engagement			
With WCIMA during 6 months after index ED visit	55 (47%)	8 (18%)	0.001
With another PCP during 6 months after index ED visit	4 (3%)	0 (0%)	0.58
With WCIMA or another PCP during 6 months after index ED visit	58 (49%)	8 (18%)	<0.001
